# Implications of insecticide resistance for malaria vector control with long-lasting insecticidal nets: evidence from health facility data from Benin

**DOI:** 10.1186/s12936-019-2656-7

**Published:** 2019-02-11

**Authors:** Filémon T. Tokponnon, Yolande Sissinto, Aurore Hounto Ogouyémi, Adicath Adéola Adéothy, Alioun Adechoubou, Télesphore Houansou, Mariam Oke, Dorothée Kinde-Gazard, Achille Massougbodji, Martin C. Akogbeto, Sylvie Cornelie, Vincent Corbel, Tessa B. Knox, Abraham Peter Mnzava, Martin J. Donnelly, Immo Kleinschmidt, John Bradley

**Affiliations:** 1 National Malaria Control Programme, Cotonou, Benin; 2 Ministry of Health, Cotonou, Benin; 3 Faculté des Sciences de la Santé de l’Université d’Abomey Calavi, Cotonou, Benin; 4 World Health Organization, Cotonou, Benin; 5 Centre de Recherche Entomologique de Cotonou (CREC), Cotonou, Benin; 6 Maladies Infectieuses et Vecteurs, Ecologie, Génétique, Evolution et Contrôle (MIVEGEC), Institut de Recherche pour le Développement (IRD), CNRS, University of Montpellier, Montpellier, France; 7 Global Malaria Programme, WHO, Geneva, Switzerland; 8 African Leaders Malaria Alliance (ALMA), Dar Es Salaam, Tanzania; 9 Department of Vector Biology, Liverpool School of Tropical Medicine, Liverpool, UK; 10 MRC Tropical Epidemiology Group, London School of Hygiene and Tropical Medicine, London, UK

**Keywords:** Malaria, Insecticide, Pyrethroid, Resistance, Vector

## Abstract

**Background:**

Insecticide-based interventions have averted more than 500 million malaria cases since 2000, but insecticide resistance in mosquitoes could bring about a rebound in disease and mortality. This study investigated whether insecticide resistance was associated with increased incidence of clinical malaria.

**Methods:**

In an area of southern Benin with insecticide resistance and high use of insecticide-treated nets (ITNs), malaria morbidity and insecticide resistance were measured simultaneously in 30 clusters (villages or collections of villages) multiple times over the course of 2 years. Insecticide resistance frequencies were measured using the standard World Health Organization bioassay test. Malaria morbidity was measured by cases recorded at health facilities both in the whole population using routinely collected data and in a passively followed cohort of children under 5 years old.

**Results:**

There was no evidence that incidence of malaria from routinely collected data was higher in clusters with resistance frequencies above the median, either in children aged under 5 (RR = 1.27 (95% CI 0.81–2.00) p = 0.276) or in individuals aged 5 or over (RR = 1.74 (95% CI 0.91–3.34) p = 0.093). There was also no evidence that incidence was higher in clusters with resistance frequencies above the median in the passively followed cohort (RR = 1.11 (0.52–2.35) p = 0.777).

**Conclusions:**

This study found no association between frequency of resistance and incidence of clinical malaria in an area where ITNs are the principal form of vector control. This may be because, as other studies have shown, ITNs continue to offer some protection from malaria even in the presence of insecticide resistance. Irrespective of resistance, nets provide only partial protection so the development of improved or supplementary vector control tools is required to reduce Africa’s unacceptably high malaria burden.

## Background

African malaria vector populations are becoming increasingly resistant to the insecticides used for malaria prevention [1–3]. This is especially true for pyrethroids, the class of insecticide used on all insecticidal bed nets. The growing inability of insecticides to kill malaria vectors is concerning because insecticide-based interventions are vital to preventing death and disease from malaria in African children. Insecticide-treated nets (ITNs) are estimated to be responsible for 78% of the 663 million clinical malaria cases averted in sub-Saharan Africa since 2001, and more than 50% of people in malaria-endemic areas in sub-Saharan Africa slept under ITNs in 2016 [4, 5].

However, it is not clear how the rise of insecticide resistance will affect the malaria burden in Africa. Mathematical models predict increased malaria incidence but real world evidence of this is lacking [6]. There are some data from malaria control programmes in South Africa, Equatorial Guinea and Sudan that suggest an impact of resistance on the malaria burden, but these examples relate to indoor residual spraying (IRS) of insecticides rather than ITNs, and in none of the cases is the evidence conclusive [7–11].

There have been no convincing examples of ITN malaria control failure due to pyrethroid resistance. Two recent trials have shown that in an areas of resistance, nets that either incorporated the synergist piperonyl butoxide (PBO) or another active ingredient in addition to a pyrethoid were more effective than conventional ITNs [12, 13]. Although the trials did not attempt to assess whether standard ITNs provide protection in an area of pyrethroid resistance, they did show that their effectiveness was inferior to that of the modified nets in these settings. It will take time to roll out a new class of nets in all areas with a similar resistance profile and in the meantime the question of whether conventional ITNs continue to provide sufficient protection from malaria is a pressing one.

The current study is part of a programme designed to address the impacts of insecticide resistance [14, 15]. Results published so far demonstrate that children who sleep under ITNs are at lower risk of malaria infection as measured by cross-sectional surveys, and that children who sleep under ITNs experience a lower rate of clinical malaria episodes as measured by active follow up [11, 16–18]. The present study assessed the impact of insecticide resistance at the community level on clinical malaria episodes severe enough to prompt a visit to a health facility for treatment. Specifically, the aim is to answer the question of whether higher frequencies of resistance to pyrethroids are associated with a greater rate of clinical malaria in an area where ITNs are the main form of vector control.

## Methods

To answer the study question, insecticide resistance and incidence of clinical malaria were measured concurrently in 30 clusters in two consecutive years to test for associations.

### Study setting

The study was conducted in the Plateau Department of Benin. Observations were made in 30 clusters (villages or collections of villages) in four rural districts: Ifangni, Sakete, Ketou, and Pobe. The area of the study region is 3264 sq km, with an approximate population of 400,000. The estimated populations of the study clusters ranged from 961 to 7496 with a median of 2132. There are two rainy seasons, one from April to July and a second from September to October. The principal vectors of malaria across the area are *Anopheles gambiae* sensu stricto and *Anopheles coluzzii* [19]. In West Africa, there is extensive introgression of resistance mutations between the two species [20, 21]. In Benin, levels of resistance in the two species were found to be similar [20], and as such the two species are treated as a single entity for the purpose of this study. ITNs are the main form of vector control in the area. The national malaria control programme distributed ITNs (a mixture of PermaNet^®^ 2.0 (Vestergaard), Olyset^®^ Net (Sumitomo), and DawaPlus^®^ 2.0 (Tana Netting)) in 2011 and 2014. In 2015, 78% of children under 5 were reported to have used an ITN the previous night [16].

### Entomological measurements

Mosquito larvae were collected from aquatic habitats in each cluster and were reared to adults to assess susceptibility to pyrethroids. Sampling took place three times during the study: June 2013, October 2013 and July 2014. Larvae were reared until they were 2–5 days-old adults. Female mosquitoes were exposed to the discriminating dose of deltamethrin (0.05%) using the standard World Health Organization (WHO) bioassay test [22] at a relative humidity of 80 ± 10% and a temperature of 25 ± 2 °C mosquito mortality was recorded 24 h after deltamethrin exposure.

### Malaria morbidity measurements

Estimates of cluster level malaria morbidity came from two independent sources. First, registers of patients routinely kept at health facilities for 2013 were examined. Data on the number of malaria cases with axillary temperature above 37.5 °C and confirmed by either microscopy or rapid diagnostic test were recorded from health facilities in the study area. The registers contained information about where each case lived so it was possible to determine whether the case came from one of the study clusters. It was also recorded whether the case was aged under 5. Estimates for the population of each cluster taken from a census in 2012 were used to calculate the person-time at risk of clinical malaria in each cluster. Second, at the beginning of 2013, 70 children aged under 5 in each cluster were recruited into a cohort. Each child was given a card entitling them to free health care at the local health facility. Each visit to a health facility by a child carrying a study card was recorded. The record included information on the result of any malaria diagnosis by rapid diagnostic test (RDT) or microscopy. Follow up was from January 2013 to December 2014.

### Sample size

For the routine health facility data, 30 clusters with median size of 380 children under 5 per cluster observed for 1 year would give 93% power to detect a difference in the rate of clinical malaria between the 15 highest resistance clusters and the 15 lowest resistance clusters if there is a rate of 0.2 cases per child year in the highest resistance clusters and a 33% lower rate of 0.13 cases per child year in the lowest resistance clusters, assuming a coefficient of between cluster variation of 0.25.

For the passively followed cohort, 30 clusters with 70 children per cluster followed for 2 years would give 90% power to detect a difference in the rate of clinical malaria between the 15 highest resistance clusters and the 15 lowest resistance clusters if there is a rate of 0.2 cases per child year in the highest resistance clusters and a 33% lower rate of 0.13 cases per child year in the lowest resistance clusters, assuming a coefficient of between-cluster variation of 0.25.

### Statistical analysis

Although mosquito mortality was measured at three time points in the study period, child follow up was continuous. To account for this, child follow-up time was divided into three periods and each period was matched to one of the three measurements of mosquito mortality. Follow up from January to July 2013 was matched with the mosquito mortalities in June 2013; August to December 2013 was matched with the mosquito mortality measurements taken in October 2013; and (for the passively followed cohort) January to December 2014 was matched with the mosquito mortality measurements taken in July 2014. During each study period clusters were classified as higher resistance or lower resistance according to whether mosquito mortality was below or above the median, respectively. Analyses were performed both separately in each time period and for the total combined follow up (for the combined follow up, higher resistance was defined in terms of the median of all measurements). For the routine health centre data, analyses were performed separately for children aged under 5 and for people aged 5 or over.

In each time period, malaria incidence was estimated as the number of incident cases per child-year of follow up. In the passively followed cohort, a 2-week period was subtracted from the time at risk to account for the prophylactic effect of the treatment. Incidence was calculated overall and separately for higher and lower resistance clusters.

Poisson regression was used to estimate the incidence rate ratio (RR) between higher and lower resistance clusters. Poisson regression was also used to estimate the linear effect of a reduction in mosquito mortality on cluster-level clinical malaria incidence. Robust standard errors were used to account for correlation of responses within clusters.

For the passively followed cohort there were a small number of highly influential outlying observations. Analyses were repeated omitting clusters where the rate was rate greater than 2 standard deviations above the mean cluster-level rate.

## Results

### Insecticide resistance

Insecticide resistance was evaluated in all 30 clusters over the 2 years of the study: (n = 26 June 2013, n = 27 October 2013, n = 26 July 2014). Twenty-one clusters had resistance measured at all three time points. A total of 7388 mosquitoes were tested for resistance: 1975 in June 2013 (median of 77 per cluster); 2955 in October 2013 (median of 109 per cluster); and, 2458 in July 2014 (median of 94 per cluster).

**Fig. 1:**
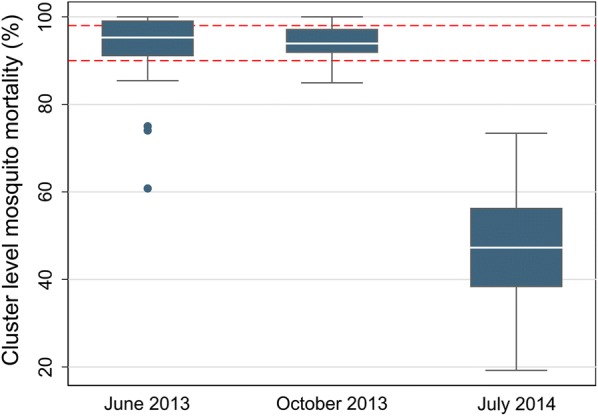
Cluster level malaria mosquito mortality on exposure to deltamethrin. The red dotted lines represent 98% mortality (possible resistance) and 90% mortality (confirmed resistance)

Median cluster level mortality following deltamethrin exposure was 95.3% in June 2013, 93.9% in October 2013 and 47.3% in July 2014 (Fig. 1). Of the 79 mortality observations, 13 (16%) were above 98% mortality (susceptible using the WHO classification (22)), 31 (39%) were between 98 and 90% mortality (possible resistance), and 35 (44%) were below 90% mortality (confirmed resistance) (Fig. 1).

### Health facility data

**Fig. 2:**
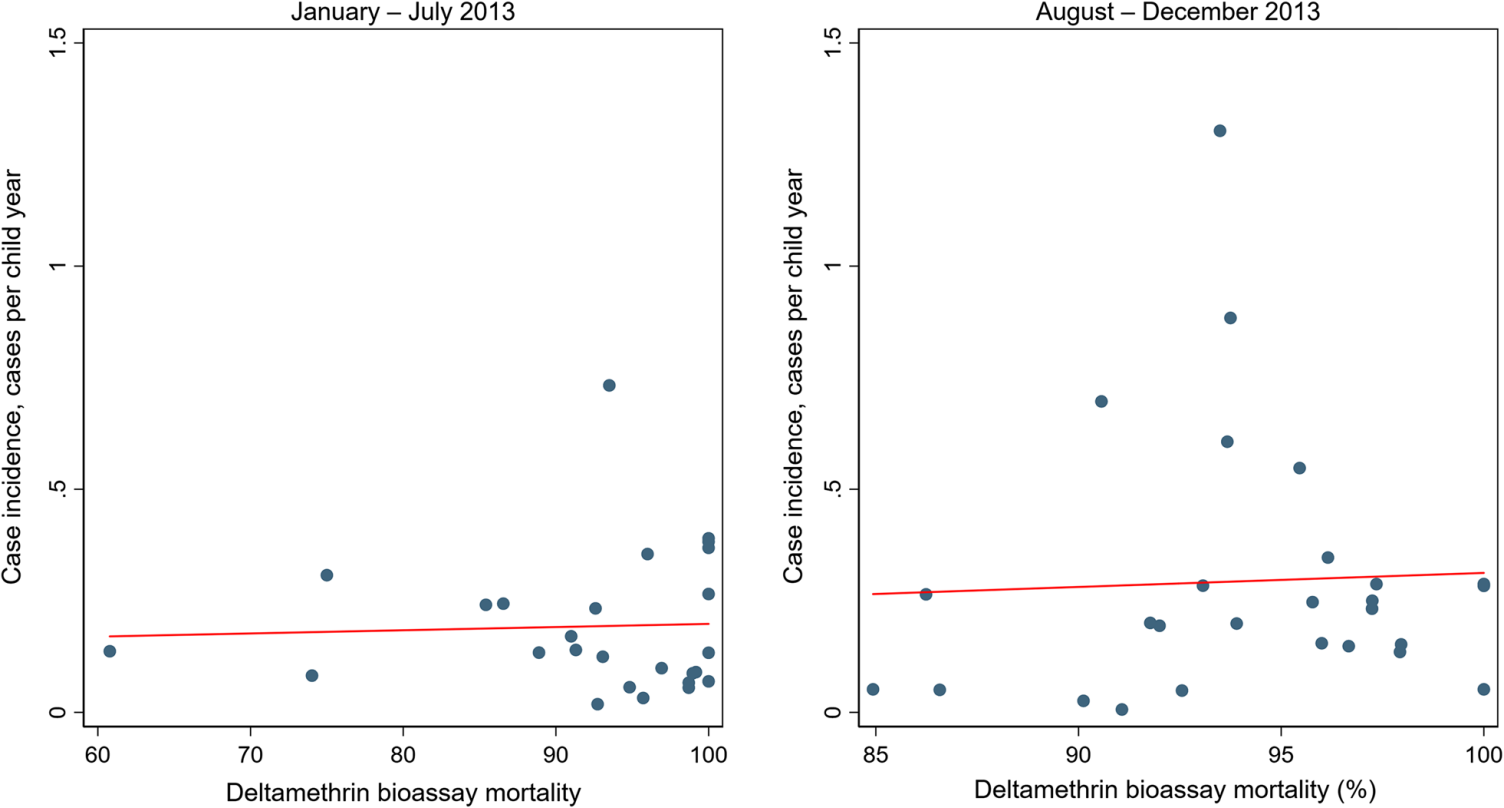
Cluster level malaria mosquito mortality on exposure to deltamethrin versus rate of clinical malaria in children under 5 taken from health facility data from January to July 2013 (left) and August to December 2013 (right). Red lines represent lines of best fit

For children under 5 there were 3108 confirmed cases of malaria in 2013 at a rate of 0.24 cases per child-year (95% CI 0.18–0.32) (Table 1). There was no evidence that the rate was higher in higher resistance clusters in 2013 as a whole (RR = 1.27 (95% CI 0.81–2.00) p = 0.276) nor was there evidence of higher incidence in higher resistance clusters either in January to July (RR = 1.33 (95% CI 0.70–2.52) p = 0.368) or in August to December (RR = 1.12 (95% CI 0.63–2.02) p = 0.684). There was no evidence of a linear association at either time period (Fig. 2).

**Table 1:** Rates of clinical malaria from routine health facility data

	Under 5 years of age		5 years of age or over	
	Malaria incidence per person-year (95% CI) [cases/person-years]	Rate ratio (95% CI)	Malaria incidence per person-year (95% CI) [cases/person-years]	Rate ratio (95% CI)
*January–July 2013*				
Overall	0.20 (0.15–0.26) [1545/7832]	–	0.023 (0.016–0.034) [1015/43,510]	–
Lower resistance (mortality equal to or above median value of 95.3%)	0.18 (0.11–0.30) [635/3443]	1	0.016 (0.010–0.028) [311/19,126]	1
Higher resistance (mortality below median value of 95.3%)	0.21 (0.14–0.31) [910/4389]	1.12 (0.63–2.02) p = 0.684	0.029 (0.017–0.049) [704/24,385]	1.77 (0.88–3.56) p = 0.102
Effect per 10% reduction in bioassay mortality	–	1.02 (0.80–1.29) p = 0.863	–	1.06 (0.85–1.32) p = 0.605
*August–December 2013*				
Overall	0.30 (0.21–0.42) [1563/5211]	–	0.030 (0.019–0.049) [879/28,948]	–
Lower resistance (mortality equal to or above median value of 93.9%)	0.26 (0.19–0.35) [728/2798]	1	0.024 (0.017–0.033) [370/15,545]	1
Higher resistance (mortality below median value of 93.9%)	0.35 (0.19–0.64) [835/2413]	1.33 (0.70–2.52) p = 0.368	0.038 (0.017–0.087) [509/13,403]	1.60 (0.69–3.68) p = 0.261
Effect per 10% reduction in bioassay mortality	–	0.94 (0.50–1.77) p = 0.854	–	0.89 (0.43–1.81) p = 0.731
*January–December 2013 (both time periods combined)*				
Overall	0.24 (0.18–0.32) [3108/13,044]	–	0.026 (0.017–0.039) [1894/72,458]	–
Lower resistance (mortality equal to or above median value of 93.9%)	0.21 (0.16–0.28) [1376/6472]	1	0.019 (0.014–0.026) [693/35,952]	1
Higher resistance (mortality below median value of 94.8%)	0.26 (0.17–0.41) [1732/6572]	1.27 (0.81–2.00) p = 0.276	0.033 (0.018–0.060) [1201/36,506]	1.74 (0.91–3.34) p = 0.093
Effect per 10% reduction in bioassay mortality	–	1.01 (0.82–1.25) p = 0.915	–	1.04 (0.86–1.26) p = 0.677

**Fig. 3:**
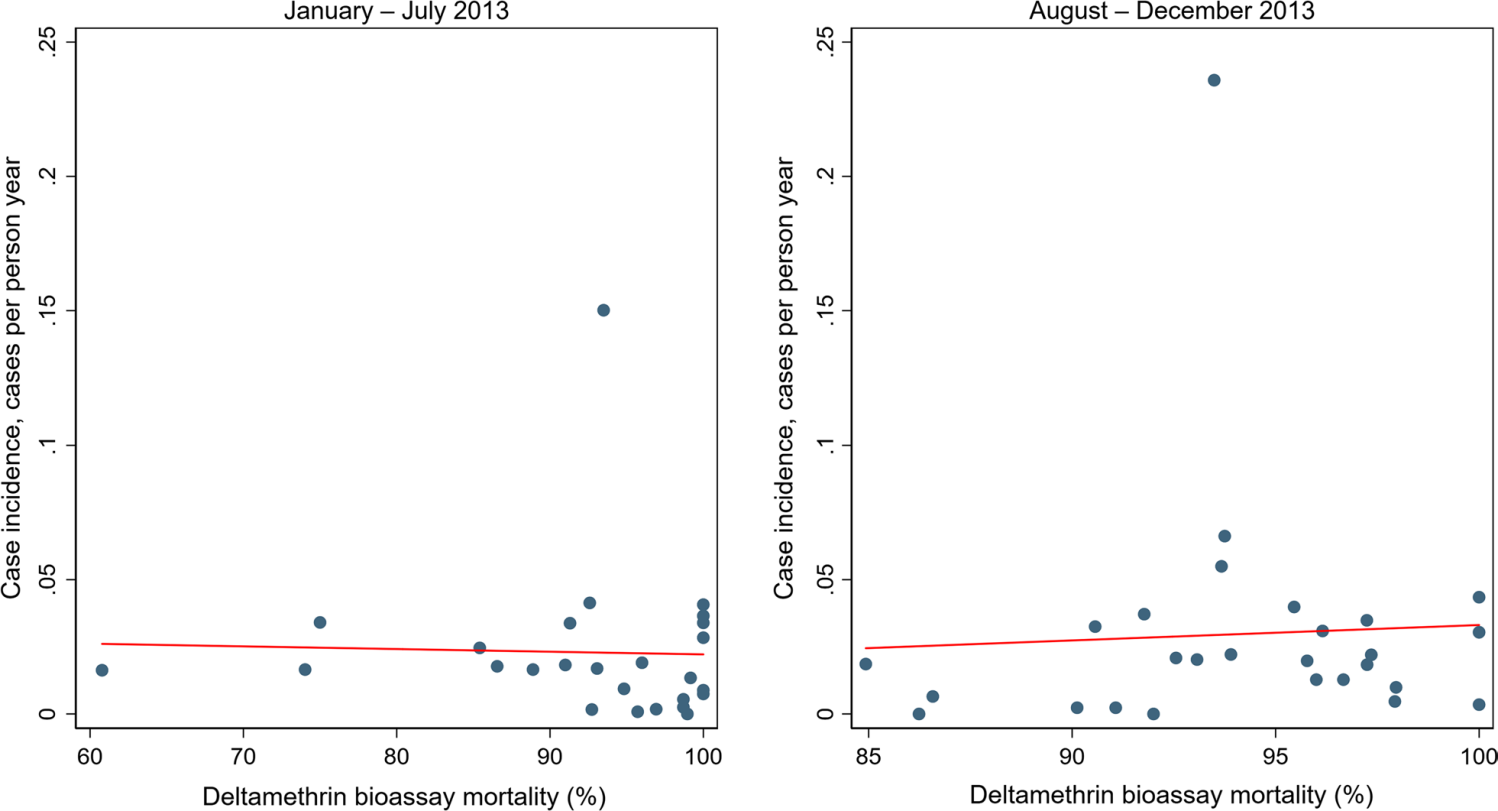
Cluster level malaria mosquito mortality on exposure to deltamethrin versus rate of clinical malaria in individuals aged 5 or over taken from health facility data from January to July 2013 (left) and August to December 2013 (right). Red lines represent lines of best fit

For those aged 5 or over, there were 1894 confirmed cases of malaria in 2013 at a rate of 0.026 cases per person year (95% CI 0.017–0.039) (Table 1). Similarly as for under 5 s, there was no evidence that the incidence rate was higher in higher resistance clusters over the whole of 2013 (RR = 1.74 (95% CI 0.91–3.34) p = 0.093). There was also no evidence of higher incidence in higher resistance clusters either in January to July (RR = 1.77 (95% CI 0.88–3.56) p = 0.102) or in August to December (RR = 1.60 (95% CI 0.69–3.68) p = 0.261). There was no evidence of a linear association in either time period (Fig. 3).

### Passively followed cohort

**Fig. 4:**
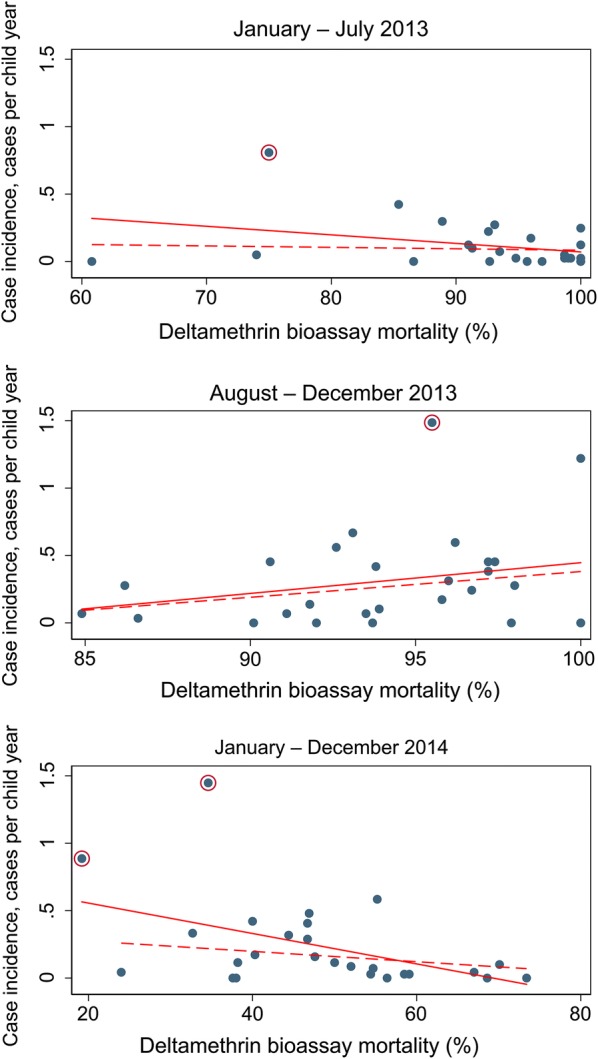
Cluster level malaria mosquito mortality on exposure to deltamethrin versus rate of clinical malaria from a passively followed cohort of children under 5 years of age from January to July 2013 (top), August to December 2013 (middle), and January to December 2014 (bottom). Solid red lines represent lines of best fit; dashed red lines represent lines of best fit omitting outliers (these are the circled points defined as cluster level rate greater than 2 standard deviations above the mean cluster-level rate)

There were 784 confirmed clinical malaria cases in the 2 years of follow up at a rate of 0.22 cases per child year (95% CI 0.14–0.34) (Table 2). There was no evidence that the rate of clinical malaria was higher in clusters with higher resistance (RR = 1.11 (95% CI 0.52–2.35) p = 0.777). There was evidence that there was higher incidence in higher resistance clusters in January to July 2013 (RR = 3.45 (95% CI 1.17–10.20) p = 0.027) and January to December 2014 (RR = 3.93 (95% CI 1.34–11.51) p = 0.015). However, when outlying clusters with rates greater than 2 standard deviations above the mean cluster-level rate were removed (1 in January to July 2013 and 2 in January to December 2014) the effects were no longer significant (Fig. 4).

**Table 2:** Rates of clinical malaria from a passively followed cohort of children under 5 years of age

	Malaria incidence per person-year (95% CI) [cases/person-years]	Rate ratio (95% CI)	Rate ratio excluding outliers^a^ (95% CI)
*January–July 2013*			
Overall incidence	0.12 (0.06–0.22) [124/1057]	–	–
Lower resistance (mortality equal to or above median value of 95.3%)	0.05 (0.02–0.13) [28/530]	1	1
Higher resistance (mortality below median value of 95.3%)	0.18 (0.09–0.39) [96/527]	3.45 (1.17–10.20) p = 0.027	2.48 (0.88–7.00) p = 0.082
Effect per 10% reduction in bioassay mortality	–	1.46 (0.84–2.54) p = 0.170	1.11 (0.68–1.82) p = 0.667
*August–December 2013*			
Overall incidence	0.31 (0.19–0.49) [240/778]	–	–
Lower resistance (mortality equal to or above median value of 93.9%)	0.42 (0.22–0.80) [158/373]	1	1
Higher resistance (mortality below median value of 93.9%)	0.20 (0.11–0.39) [82/405]	0.48 (0.21–1.11) p = 0.083	0.60 (0.26–1.37) p = 0.214
Effect per 10% reduction in bioassay mortality	–	0.45 (0.16–1.33) p = 0.143	0.46 (0.14–1.58) p = 0.208
*January–December 2014*			
Overall incidence	0.23 (0.13–0.41) [420/1804]	–	–
Lower resistance (mortality equal to or above median value of 47.2%)	0.09 (0.04–0.25) [86/907]	1	1
Higher resistance (mortality below median value of 47.2%)	0.37 (0.20–0.70) [334/897]	3.93 (1.34–11.51) p = 0.015	2.46 (0.89–6.78) p = 0.080
Effect per 10% reduction in bioassay mortality	–	1.61 (1.21–2.13) p = 0.002	1.28 (0.91–1.79) p = 0.144
*January 2013–December 2014 (all time periods combined)*			
Overall incidence	0.22 (0.14–0.34) [784/3639]	–	–
Lower resistance (mortality equal to or above median value of 47.2%)	0.20 (0.13–0.31) [270/1378]	1	1
Higher resistance (mortality below median value of 91.3%)	0.23 (0.13–0.40) [514/2261]	1.11 (0.52–2.35) p = 0.777	0.89 (0.42–1.86) p = 0.738
Effect per 10% reduction in bioassay mortality	–	1.50 (1.14–1.97) p = 0.005	1.17 (0.90–1.53) p = 0.239

^a^ Defined as cluster level rate greater than 2 standard deviations above the mean cluster-level rate

Whilst there was evidence of a linear association between increasing resistance and clinical incidence during the 2 years of follow up RR = 1.50 for a 10% decrease of mosquito mortality (95% CI 1.14–1.97, p = 0.005) when outlying clusters with rates greater than 2 standard deviations above the mean cluster-level rate were removed, the association was no longer significant RR = 1.17 (95% CI 0.90–1.53, p = 0.239).

## Discussion

This study found no evidence of an association between vector resistance and incidence of clinical malaria measured by routine health facility data in an area where ITNs were the principal form of malaria control. This was true for children aged under 5 and for those aged over 5. There was some evidence that incidence of clinical malaria in a passively followed cohort was higher in higher resistance, but this evidence was not robust and was the result of a small number of outlying clusters; once these clusters were removed, there was no evidence of an association.

This study is in line with the results of the same network of studies on the impact of insecticide resistance which have reported on less severe outcomes than this study [11, 16–18]. All other studies in the network, as well as other studies [23], indicate that ITNs continue to provide protection with no association found between resistance and malaria outcomes including malaria prevalence, infection incidence, and actively detected clinical malaria incidence. Therefore, this study is consistent with previous findings that looked at less severe outcomes.

Why might there be no association between vector resistance and incidence of clinical malaria in an area where ITNs are the principal method of vector control? Firstly, even without insecticide, nets are a physical barrier between the sleeper and the mosquito. Secondly, resistance is not an all-or-nothing phenomenon; even if the proportion of mosquitoes killed within 24 h of exposure is reduced, the insecticide may still have an impact. For example, a meta-analysis found that ITNs gave greater protection than untreated nets [24], even in areas where mosquitoes were resistant to pyrethroids. There may also be more subtle effects in play. Oocyst development may be slower in resistant mosquitoes exposed to a pyrethroid compared to those that have not been exposed [25], and there may be post-24-h mortality when pyrethroid-resistant malaria vectors are exposed to deltamethrin [26].

This study used two different methods to estimate the rate of clinical malaria severe enough to prompt a visit to a health facility. If the two methods gave very different estimates it would have cast doubt on the validity of the study. Routine health records gave an estimate of 0.22 cases per child year for children aged under 5 (95% CI 0.14–0.34) and the passive cohort gave a very similar estimate of 0.20 cases per child year for children aged under 5 (95% CI 0.13–0.30). An actively followed cohort in the same clusters gave an estimate of 0.48 cases per child year for children aged under 5 (95% CI 0.39–0.59). One would expect the rate to be higher in the actively followed cohort because the follow up would detect cases of malaria that might not have been severe enough to prompt a visit to a health facility.

This study had several limitations. Firstly, there is no direct observation of ITNs providing protection. Instead, it must be inferred at the ecological level from the fact that even though ITNs are the primary form of malaria control in the study area, malaria incidence was not higher in villages where resistance was higher. The passive nature of the study meant that it was not possible to record ITN use and provide an estimate of the level of individual protection afforded by ITNs. Furthermore, aside from personal protection the ITNs may also provide protection through mass effect, whereby the killing of mosquitoes reduces local mosquito longevity and density and provides protection for all members of the community, including those who do not use ITNs. The study design used did not enable differentiation between these two types of protection.

A second limitation is that the range of insecticide measurements was small in 2013, with the majority of clusters between 90 and 98% mortality (defined by the WHO as possible resistance) with relatively few clusters classified as harbouring vectors with confirmed resistance (< 90% mortality). Insecticide resistance measurement was based on the WHO insecticide susceptibility test, measuring simply the frequency of resistant mosquitoes. Measures of resistance intensity based on a dose–response relationship which capture the intensity of resistance may been more informative, but were not practical for the scale of this study and the other associated studies [27].

Thirdly, in some clusters study staff were unable to find larvae at every time point so there are a small number of missing resistance measurements. This study made the assumption that mosquitoes collected at larval stage are representative of the population transmitting malaria at the adult stage. This is a reasonable assumption given that *An. gambiae s.l.* is the predominant anopheline collected at both larval and adult stages and has been implicated as the primary malaria vector in Plateau State [15].

Fourthly, randomization of locations to insecticide resistance is not possible. Therefore, any study on this issue must have an observational design, rendering it subject to confounding factors. The nature of this study meant it was not possible to record potential confounding factors such as socio-economic status. Nevertheless, studies that did control for confounding factors found similar results to the current study for less severe malaria outcomes [16, 17].

## Conclusion

This study did not find evidence for an association between insecticide resistance and malaria incidence in an area reliant on ITNs for malaria control. This is in accordance with the results of previous studies that looked at less severe malaria outcomes. Taken together with other studies in this programme, these results provide additional evidence that, even in the presence of insecticide resistance, populations living in malaria-endemic areas are protected from malaria by pyrethroid-only ITNs and, therefore, their use should continue. Nevertheless, new tools such as new generation nets that are not solely reliant on pyrethroids will be essential to sustain and further the gains made against malaria. In the interim, efforts should be made to increase access to good condition long-lasting insecticidal nets.

## Abbreviations

ITN: insecticide-treated net
IRS: indoor residual spraying
PBO: piperonyl butoxide
WHO: World Health Organization
RDT: rapid diagnostic test
CI: confidence interval
RR: rate ratio
